# Use of a blood gas analyzer and a laboratory autoanalyzer in routine practice to measure electrolytes in intensive care unit patients

**DOI:** 10.1186/1471-2253-12-17

**Published:** 2012-08-03

**Authors:** Yasemin U Budak, Kagan Huysal, Murat Polat

**Affiliations:** 1 Department of Clinical Laboratory, Sevket Yilmaz Education and Research Hospital, Sevket Yilmaz Devlet Hastanesi, Biyokimya Laboratuari Yildirim, Bursa, Turkey; 2 Department of Clinical Laboratory, Yüksek İhtisas Education and Research Hospital, Bursa, Turkey; 3 Department of General Surgery, Sevket Yilmaz Education and Training Hospital, Bursa, Turkey

## Abstract

**Background:**

Electrolyte values are measured in most critically ill intensive care unit (ICU) patients using both an arterial blood gas analyzer (ABG) and a central laboratory auto-analyzer (AA). The aim of the present study was to investigate whether electrolyte levels assessed using an ABG and an AA were equivalent; data on sodium and potassium ion concentrations were examined.

**Methods:**

We retrospectively studied patients hospitalized in the ICU between July and August 2011. Of 1,105 test samples, we identified 84 instances of simultaneous sampling of arterial and venous blood, where both Na^+^ and K^+^ levels were measured using a pHOx Stat Profile Plus L blood gas analyzer (Nova Biomedical, Waltham MA, USA) and a Roche Modular P autoanalyzer (Roche Diagnostics, Mannheim, Germany). Statistical measures employed to compare the data included Spearman's correlation coefficients, paired Student’s *t*-tests, Deming regression analysis, and Bland-Altman plots.

**Results:**

The mean sodium concentration was 138.1 mmol/L (SD 10.2 mmol/L) using the ABG and 143.0 mmol/L (SD 10.5) using the AA (p < 0.001). The mean potassium level was 3.5 mmol/L (SD 0.9 mmol/L) using the ABG and 3.7 mmol/L (SD 1.0 mmol/L) using the AA (p < 0.001). The extent of inter-analyzer agreement was unacceptable for both K^+^ and Na^+^, with biases of 0.150-0.352 and −0.97-10.05 respectively; the associated correlation coefficients were 0.88 and 0.90.

**Conclusions:**

We conclude that the ABG and AA do not yield equivalent Na^+^ and K^+^ data. Concordance between ABG and AA should be established prior to introduction of new ABG systems.

## Background

Electrolytes are charged elements that are essential for proper cellular functioning in most tissues of the body. Almost all metabolic processes are dependent upon or are mediated by electrolytes. Variation in electrolyte concentrations may be either the cause or the consequence of a variety of disorders; such problems must be identified to ensure adequate treatment. Electrolyte abnormalities can represent significant risks to life [1].

In the intensive care unit (ICU), electrolyte values are measured in most critically ill patients [2]. Under such circumstances it is important to obtain data quickly so as to optimize the therapeutic response interval and allow prompt treatment.

Two methods of electrolyte assay, one direct and one indirect, both employing ion-sensing electrodes (ISEs), are currently in use in most hospitals [3]. The indirect assay features pre-analytic dilution and is often employed in high-throughput central hospital laboratories running automated analyzers (AAs) [3]. In the direct ISE method, the electrode surface contacts a complete undiluted blood sample; this approach is employed by arterial blood gas analyzers (ABGs) or point-of-care testing (POCT) equipment [3]. Indirect ISE devices use diluted plasma (or serum) samples; the results are generally comparable to those afforded by flame photometry (the recognized reference method) [4]. Sodium and potassium levels measured in whole blood and plasma have been shown to be essentially identical [5].

The use of central laboratory testing in a hospital may cause a long delay between the time at which a test is ordered and the time at which the result is received by a clinician; such delays may compromise the treatment of critically ill patients [6]. If analysis is performed in the ICU several processing steps can be eliminated, results are obtained rapidly, patient management is timely, and outcomes improve. Of course, such advantages are possible only if the analytical performance of ICU-based tests is acceptable in comparison with those of central laboratory methods, and if the desired clinical criteria are met [7].

ABG use is rising, particularly in ICUs, emergency departments, and operating theatres; physicians frequently rely on ABG test data but send an additional sample to the central laboratory. The apparently tandem use of ABG and central laboratory analyzers to measure electrolytes increases the variability of test results; the reliability and validity of such data require examination.

In the present study we explored whether sodium and potassium ion concentrations measured with an ABG and an AA were equivalent.

## Methods

We conducted a retrospective study on data contained in the Şevket Yilmaz Research and Education Hospital Clinical Data Warehouse, a centralized data repository integrating information in several databases including the order entry database and the laboratory results database of our hospital. Prescription data are linked to detailed clinical information including patient demographics, diagnosis, and laboratory data; the latter include specimen collection date, time, and location (for example: ICU).

### Study population

The present study was approved by the Ethics Committee of the Bursa Sevket Yilmaz Research and Educational Hospital. All procedures were in accordance with the Second Declaration of Helsinki.

We studied patients who had been hospitalized in the ICU for some time in the interval between July and August 2011. We identified 84 instances, of 1,105 patient blood gas samples analyzed, in which arterial and venous samples were collected simultaneously and Na^+^ and K^+^ were measured using two methods. In the ICU, arterial blood samples were collected in heparinized blood–gas syringes (Gaslyte, Totawa, NJ) and analyzed using a benchtop blood–gas analyzer (pHOx Stat Profile Plus L, Nova Biomedical, Waltham MA, USA) which employs direct ISE technology. The blood gas analyzer was calibrated with the aid of a Nova Biomedical calibrator pack provided by the supplier, in line with NIST standards.

We identified patients from whom a further sample was drawn, at the same time, from the same arterial sampling point, using a vacuum technique featuring clot-activating tubes (Green-Vac, Yongin, Korea); the samples were sent, pneumatically sealed, to our central laboratory, where serum Na^+^ and K^+^ concentrations were analyzed via indirect ISE on a Roche Modular ISE 900 (Roche Diagnostics, Mannheim, Germany).

### Analytical precision of Na^+^ and K^+^ determinations

Before data analysis, we ran a two-level quality-control (QC) test using materials supplied by the manufacturers of both devices (Stat Profile pHOx Plus Control 1, 2; Lots 011004 and 011005, and PreciControl ClinChem Multi 1; Lots 158565 and 158577). The reproducibility of results obtained throughout the study was evaluated via analysis of duplicate QC samples on each of 20 days (between-day differences were calculated) (Table 1). For quality assurance purposes, our laboratory participates in the Riqas external quality assessment scheme; Cycle 8 (Samples 7–8) ran during the study interval. The mean comparative K^+^ level (instrument group mean) was 4.195 mmol/l whereas our figure was 4.2 mmol/l; the mean K^+^ level was 6.04 mmol/l whereas our figure was 6.35 mmol/l; the mean Na^+^ level was 143.71 mmol/l whereas our figure was 144.0 mmol/l; and the mean comparative Na^+^ level (instrument group mean) was 157.18 mmol/l whereas our figure was 155 mmol/l.

**Table 1:** The between-run precision of electrolyte assay data were determined via analysis of duplicate quality control materials on each of 20 days

**Electrolyte (mmol/L)**	**AA**	**%CV**	**ABG**	**%CV**
	**Mean**		**Mean**	
**Sodium**	140.3	1.36	157.0	1.36
	115.7	2.12	133.6	2.12
**Potassium**	6.03	1.67	5.60	1.67
	3.47	2.76	3.79	2.76

### Statistical methods

Data were evaluated using SPSS version 13.0 (SPSS Inc., Chicago, IL) and Analyse-It version 2.04 (Analyse-It Software, Leeds, UK). Data were tested for normality using the Kolmogorov-Smirnov test. Means, standard deviations, and coefficients of variation were calculated. Deming regression analysis was performed and Bland–Altman plots were constructed to compare the results of the two methods [8]. p < 0.001 was considered statistically significant.

## Results

The mean sodium level measured on the ABG was 138.1 mmol/L (SD 10.2 mmol/L) and the value obtained using the AA was 143.0 mmol/L (SD 10.5 mmol/L). A significant difference was evident when the mean (±SD) sodium levels yielded by the ABG and AA were compared (p < 0.001). The maximum difference in sodium level was 12 mmol/L and the minimum 0 mmol/L. The mean difference was 4.9 mmol/L with an SD of 3.0 mmol/L (p < 0.001). As a significant difference was detected, the null hypothesis was rejected. The correlation coefficient (the r^2^ value) was 0.90. The adjusted r^2^ value was associated with a 95% confidence interval of 0.90-0.94.

**Figure 1:**
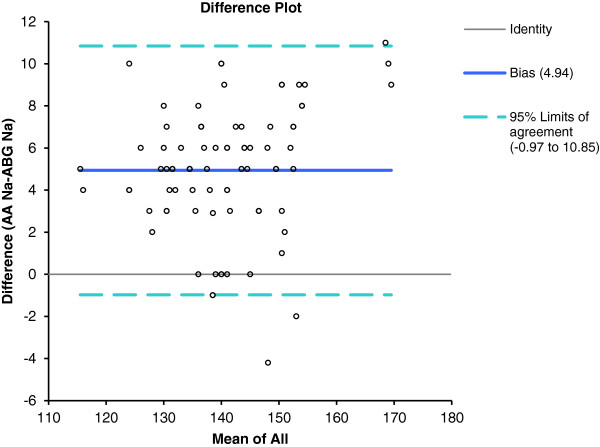
Bland-Altman plot of AA and ABG blood Na + showing the 95% limits of agreement.

A Bland–Altman comparison of central laboratory AA data with ICU ABG Na^+^ measurements showed that the limits of agreement were minus 0.97 to 10.05 mmol/L (Figure 1).

**Figure 2:**
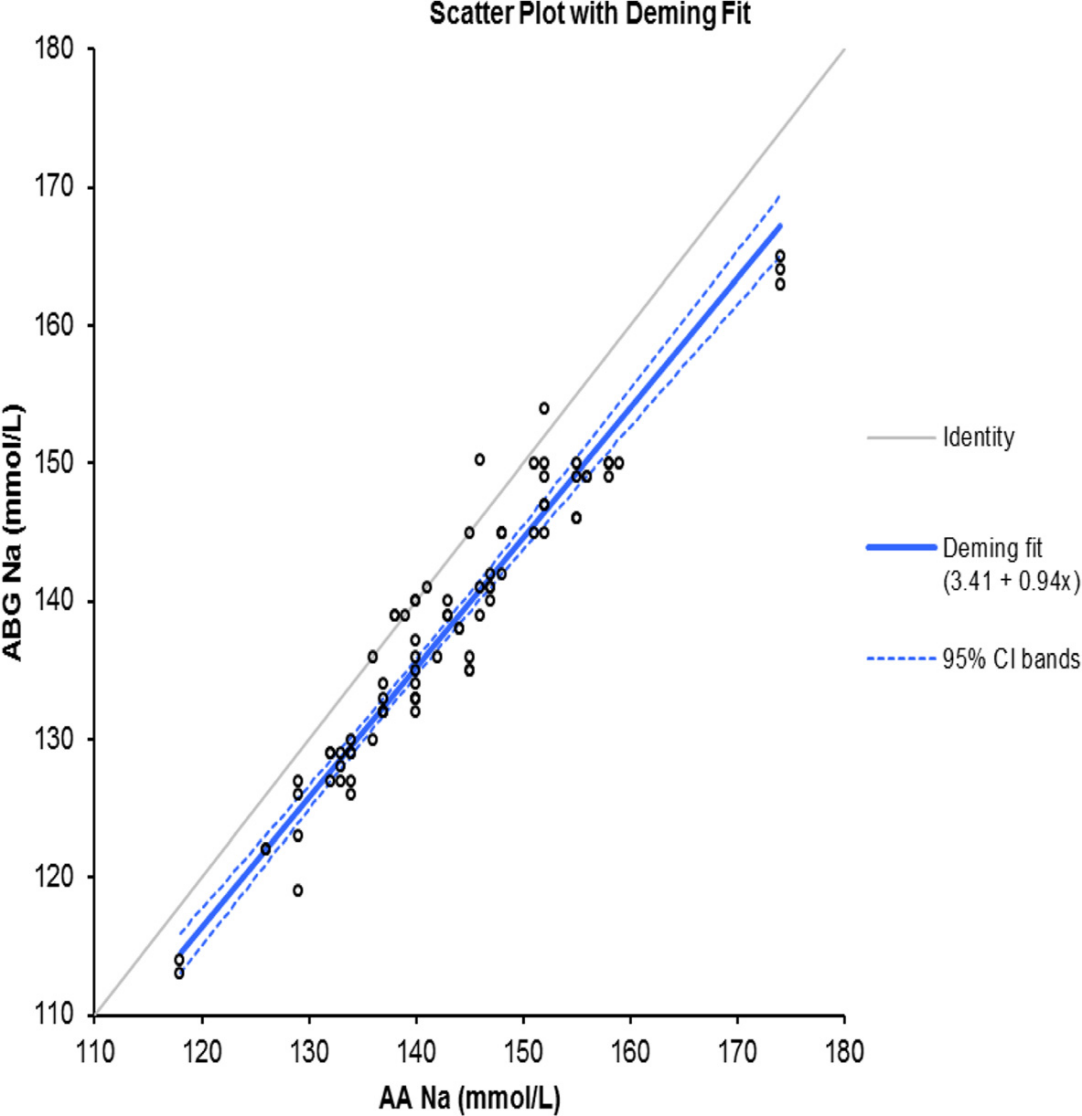
Deming fit (solid black line) with 95% confidence intervals for the ABG Na results vs laboratory (AA) Na results.

Deming regression analysis of the ABG and AA data on Na^+^ levels yielded the following formula: [ABG Na^+^ (mmol/L) = 3.41 + 0.94 AA Na^+^ (mmol/L)] (Figure 2).

**Figure 3:**
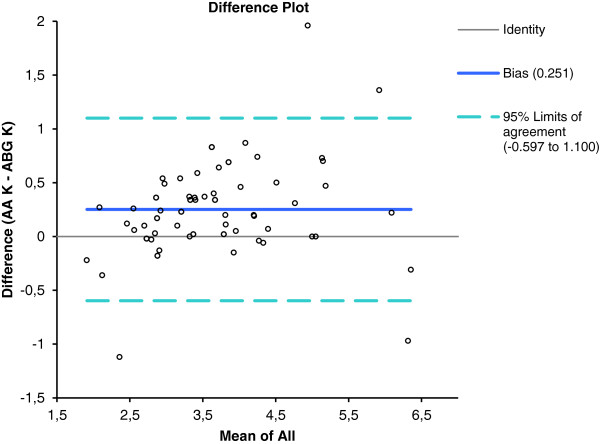
Bland-Altman plot of AA and ABG blood K + showing the 95% limits of agreement.

**Figure 4:**
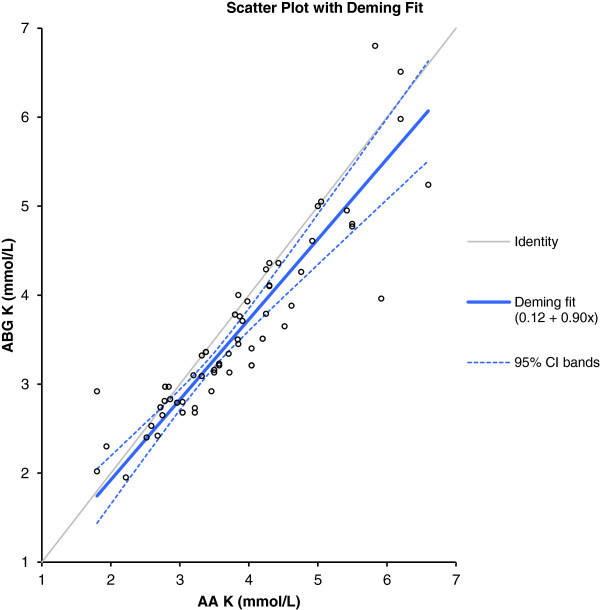
Deming fit (solid black line) with 95% confidence intervals for the ABG K + results vs laboratory (AA) K + results.

Analysis of the potassium levels measured using the ABG and the AA yielded a mean difference of 0.25 mmol/L with a SD of 0.43 mmol/L. A significant difference was evident (p < 0.001; the null hypothesis was thus rejected) between K^+^ levels measured by the ABG (3.5 mmol/L, SD 0.9 mmol/L) and the AA (mean 3.7 mmol/L, SD 1.0 mmol/L). The maximum difference in measured potassium value was 1.96 mmol/L, and the minimum 0 mmol/L (Figure 3). The correlation coefficient (the r^2^ value) was 0.88. The adjusted r^2^ value was associated with a 95% confidence interval of 0.81-0.92. Deming regression analysis of the ABG and AA data on K^+^ levels yielded the following formula: [ABG K^+^ (mmol/L) = 0.12 + 0.90 AA K^+^] (Figure 4).

Bland–Altman comparison of the AA and ABG K^+^ data yielded limits of agreement of 0.150 and 0.352 mmol/L.

## Discussion

In the present study we investigated whether Na^+^ and K^+^ levels measured using different methods and equipment, namely an ABG and an AA, were equivalent. If so, the data could be employed interchangeably in routine practice.

To ensure the accuracy of test results, our central laboratory (employing an AA) participates in an external quality assessment (EQA) program; both electrolytes were assayed with reasonable accuracy during the study period. However, the accuracy of ABG data was not evaluated via any EQA program; this is an important limitation of the present study.

The between-day imprecision of both instruments (AA and ABG) was small and lacked clinical significance when compared with analytical performance indicators based on biological variation [9] or with the United States Clinical Laboratory Improvement Amendments (US CLIA) 88 performance rules [10].

Data from the ABG appeared to be correlated with AA results (r^2^ = 0.88 for K^+^ and 0.90 for Na^+^); the strength of the relationships between the two variables was acceptable.

However, biological variations in electrolyte levels are so small that a slight error will cause patients to be misdiagnosed [9]. The US CLIA 1988 rules accept a difference of 0.5 mmol/L in potassium level, and 4 mmol/L in sodium level, compared to target values [10]. In our present study; the mean difference between the two Na^+^ assays was 4.9 mmol/L; this exceeded the acceptable value of 4 mmol/L and the 95% limits of agreement of the difference were minus 0.97 and 10.05 mmol/L.

Our data are in line with those of previous studies [11-14] showing that Na^+^ values obtained using two different types of measurement differ significantly, and to an extent that may affect therapeutic choice. Our patients were critically ill in the intensive care unit (ICU). Chow et al. [14] reported that direct ISE sodium and potassium figures were lower than those obtained using indirect ISE. This is associated with the low blood protein levels characteristic of critically ill patients. In such patients, direct ISE offers more accurate and consistent electrolyte results than does indirect ISE.

The mean between-assay difference in K^+^ levels was 0.25 mmol/L. Although the mean difference between the results of the two K^+^ assays was within the range given by the US CLIA 1988 guidelines [10], a difference of 0.25 mmol/l is clinically relevant when intra-individual variation is considered. When it is recalled that the intra-individual biological variation in K^+^ level has been reported to be 4.8% [15], any bias exhibited by either method did not exceed the acceptable level of inaccuracy [15]. It is important to emphasize that the cited criteria are very strict; the acceptable inaccuracy in terms of potassium measurement is only 1.8% [15]. It is likely that the observed variations in K^+^ values of paired samples are attributable to differences in sample type, thus serum or whole blood. It is well known that potassium is released from platelets during clotting [16] and it is thus not surprising that serum potassium values are higher than are whole-blood levels. The magnitude of the difference observed by us was similar to that earlier reported (0.1-0.7 mmol/L) [3]. After obtaining analytical results similar to ours, Jain et al. [11] suggested that it was safe to make clinical decisions based on serum K^+^ levels yielded by an ABG instrument. However, in 15% of our patients the errors were greater than 0.5 mmol/L; this may have implications in clinical practice.

Although the differences in electrolyte levels obtained using the two methods are sufficiently small to not raise a risk of inappropriate therapy in most instances, Morimatsu et al. [12] calculated the anion gap and the strong ion difference in critically ill patients using results obtained from a central laboratory analyzer and a POCT device; the Stewart-Figge formula was employed. The cited authors showed that the values calculated using data obtained by different methods differed significantly; clinical interpretation and consequent therapeutic decision-making could be adversely affected.

The observed differences between electrolyte levels measured using an ABG and an AA may be explained by a combination of factors, including sample transport, dilution of serum samples prior to testing (thus, the use of indirect *vs*. direct electrodes), and variations in instrument calibration [16,17]. It is known that ISE-based instruments from different manufacturers yield Na^+^/K^+^ values that differ by 2–5%; calibration of an AA using a NIST standard lowers the figures [18]. Also, it has recently been reported that the use of different types of heparin in blood gas syringes can introduce a pre-analytical bias in electrolyte concentrations. Such syringes can introduce different negative biases when the levels of positively charged ions are measured. The extent of bias differs among syringe types [19,20].

The wide intratest variability, as shown in the Bland-Altman plots, and the statistically significant mean differences in measured ion levels between the two methods, suggest that the tests do not yield equivalent data. It is possible to compensate for variation caused by known factors using a correction factor, to render data from different instruments comparable. The question is whether such compensation is appropriate. Although a correction factor featuring compensation based on variations in average values can minimize differences between the data from two analyzers in some instances [21], we cannot recommend this approach toward comparison of Na^+^ and K^+^ test results.

Our present study illustrates the importance of determining the concordance, for each individual hospital, of electrolyte values obtained by ABG and those obtained in the central laboratory. As instrument type and calibration methods may differ among hospitals, it is important that each center conducts an in-house study. Ideally, before installation of an ABG, it would be useful to carefully evaluate the clinical significance of any difference between data yielded by central laboratory devices and POCT instruments. Such an evaluation should be conducted prior to ABG installation; this was unfortunately not the case in our hospital. Individual laboratories should utilize external NIST Standard SRM 956 to verify calibrations conducted by manufacturers and to ensure that the results afforded by direct and indirect ISEs (18) do not differ to a clinically relevant extent.

A limitation of our work is that, in the absence of clinical review, we were unable to identify any dataset as containing erroneous values. It was not possible to establish whether the central laboratory or ABG values were closer to the true values for either analyte.

## Conclusions

Na^+^ and K^+^ test results obtained using an ABG and an AA differ and the data thus cannot be used interchangeably in clinical practice. Physicians need to be aware of between-assay differences to avoid potential misdiagnosis and initiation of unnecessary treatment or investigation.

## Abbreviations

ABG, Arterial Blood Gas Analyzer; AA, Autoanalyzer; CLIA, Clinical Laboratory Improvement Amendments; EQA, External quality assessment; ISE, Ion-selective electrode; ICU, Intensive Care Unit; NIST, National Institute of Standards and Technology; POCT, Point-of-care testing; QC, Quality-control.

## Competing interests

The authors declare that they have no competing interests.

## Authors’ contributions

YB was participated in the study design, the acquisition of data, helped to perform the statistical analysis, and drafted the manuscript. KH participated in the statistical design of the study, performed and made substantial contribution to the statistical analysis and interpretation of data. MP was involved with contributing data and helped critically revise the manuscript. All authors read and approved the final manuscript.

## Pre-publication history

The pre-publication history for this paper can be accessed here:

http://www.biomedcentral.com/1471-2253/12/17/prepub
